# Using a Novel Functional Brain Network Approach to Locate Important Nodes for Working Memory Tasks

**DOI:** 10.3390/ijerph19063564

**Published:** 2022-03-17

**Authors:** Weiwei Ding, Yuhong Zhang, Liya Huang

**Affiliations:** 1 College of Electronic and Optical Engineering & College of Microelectronics, Nanjing University of Posts and Telecommunications, Nanjing 210023, China; 1219023431@njupt.edu.cn; 2 College of Automation and Artificial Intelligence, Nanjing University of Posts and Telecommunications, Nanjing 210023, China; b18040421@njupt.edu.cn; 3 National and Local Joint Engineering Laboratory of RF Integration and Micro-Assembly Technology, Nanjing 210003, China

**Keywords:** working memory, functional brain network, K-order propagation number algorithm

## Abstract

Working Memory (WM) is a short-term memory for processing and storing information. When investigating WM mechanisms using Electroencephalogram (EEG), its rhythmic synchronization properties inevitably become one of the focal features. To further leverage these features for better improve WM task performance, this paper uses a novel algorithm: Weight K-order propagation number (WKPN) to locate important brain nodes and their coupling characteristic in different frequency bands while subjects are proceeding French word retaining tasks, which is an intriguing but original experiment paradigm. Based on this approach, we investigated the node importance of PLV brain networks under different memory loads and found that the connectivity between frontal and parieto-occipital lobes in theta and beta frequency bands enhanced with increasing memory load. We used the node importance of the brain network as a feature vector of the SVM to classify different memory load states, and the highest classification accuracy of 95% is obtained in the beta band. Compared to the Weight degree centrality (WDC) and Weight Page Rank (WPR) algorithm, the SVM with the node importance of the brain network as the feature vector calculated by the WKPN algorithm has higher classification accuracy and shorter running time. It is concluded that the algorithm can effectively spot active central hubs so that researchers can later put more energy to study these areas where active hubs lie in such as placing Transcranial alternating current stimulation (tACS).

## 1. Introduction

Working Memory (WM) is one type of memory that retaining information in a short time, it can somewhat reflect people’ s intelligence capability [1]. Due to rapid ageing population, memory related cognitive disorders such as Alzheimer’s disease and Attention Deficit Hyperactivity Disorder (ADHD) are getting ever increased—the deficiency of Working Memory Capacity (WMC)—demonstrates the urgency of studying its crucial properties for better understanding and confronting those disabilities [2].

Communication between neurons is the basis for cognitive activities [3,4], more and more researchers currently are employing Electroencephalogram (EEG) techniques to derive conclusions since its convenience and high-temporal resolution [5,6,7,8]. In the meantime, the researches have demonstrated that rhythm synchronization between neurons is closely related to WM [9,10,11,12,13,14]. In particular, Gevins et al. [15] discovered that the WMC increases with the rhythm synchronization of EEG in the theta band. Besides, the rhythm synchronization could be found between prefrontal and temporal areas during the working memory, which reflects the relationship between the local progression of neural electrical activity and working memory [16,17,18]. Although these aforementioned studies fairly excited academia via its introductory insights into WM mechanism, they are inflexible for gaining a deeper level understanding of the local electrode connections in WM-based tasks, this is mainly due to the fact that EEG does not have a high spatial resolution and that neural electrical activity is affected by volumetric effects during its transmission to the scalp [19].

Since WM is a complex process completed by the collaboration of various brain regions, and inter-connection among the brain regions is identical to a network [20], the construction of the brain networks shed light into this intricate and cross-disciplinary field of computation neuroscience and cognitive science [21,22]. At present, there are three main types of brain networks: structural brain networks, functional brain networks and effective brain networks [23,24,25]. Structural brain networks are mainly abstracted by structural imaging techniques to examine the anatomical configuration of connections between neurons. Functional brain networks are assembled by functional connectivity between nodes, but they do not reflect the causal correlation and belong to un-directed networks. By contrast, effective networks are directional networks constructed by directional functional connection between nodes, which can reflect the direction of information transmission. Meanwhile, the EEG-based brain network analysis can be summarized as follows: (1) pre-processing the EEG signal and extracting the wave-forms in specific frequency bands; (2) quantifying the relationships among the EEG channels, such as inter-correlation, phase synchronization in functional networks and Granger causality in effective networks; (3) verifying the network edge threshold to binarize the network; (4) extracting the network features for ensuing analysis.

There are growing number of researches on WM based on brain networks. Hwang et al. [26] surveyed the mechanisms of working memory through comparing the structural disparity of brain networks in different WM states, and the brain could reconfigure its scale organization dynamically in response to current cognitive demands. MEG found that with increasing the memory task difficulty, functional brain networks tend to move toward random networks, with higher global efficiency and lower clustering coefficient [27]. Bashivan et al. [28] who used the causal brain network set up via information entropy, noticed that the information flow rate across a primary working memory network is moderated by memory load. Although the information processing of working memory can be well reflected by analyzing brain network features, there are still problems in this process to be further addressed. For example, structural brain network requires imaging techniques to build connectivity, which complicates the process of computing network features. Network features such as network feature path length and clustering coefficients can only measure global or local characteristics of the network, but not a combination of both [29]. In terms of the working memory paradigm, the traditional working memory paradigm like N-back focuses on the matching task memory between the current state and the previous state, suitable for the spatial and image memory, but for memorizing word-type tasks, it still has some shortcomings like ignoring the process of word memorization.

To address these deficiencies above-mentioned in previous research, this article makes the following innovations: Firstly, a new WM experiment paradigm based on the French word memory is proposed, which highlights the process of memorization by constantly memorizing words and is more suitable for the study of words working memory. Then, the Phase Locking Value (PLV) between the electrodes are employed to construct a functional brain network so that the synchronous relationship of brain regions during working memory can be acquired, and based on Surrogate data method proposed by Hurtado et al. [30], the brain network binarization is achieved, it can effectively increase the signal to noise ratio [24]. Lastly, to further analyze the global and local characteristics of the brain network, a novel algorithm named Weighted K-order Propagation Number (WKPN) algorithm is proposed by our team [31]. Comparing with the other algorithms like Weight degree centrality (WDC) and Weight Page Rank (WPR), WKPN has better convergence and compensates for the fact that some algorithms ignore bridge nodes [32,33]. In a simulation of a deliberate attack on a US airport network, WKPN was able to significantly reduce the efficiency of the network in a shorter time and number of attacks compared to WDC and WPR. For this reason, this paper would adopt WKPN algorithm to explore important brain region associated with working memory, the average accuracy rate is significantly higher than algorithms of WDC and WPR.

The paper is arranged as follows: Section 2 introduces the WM experiment paradigm and the data pre-processing method. Section 3 describes the specific process of the brain network analysis method. The node’s importance of the brain network and the result of the SVM classification accuracy rate are shown in Section 4. We discuss the conclusions and limitations of this research.

## 2. Experiment

### 2.1. Experiment Paradigm

In order to elicit word working memory while reducing the impact of the experiment on the subjects’ linguistic base, we selected a language that none of the subjects had any basic background as the memory task. In order to simulate the process of memorizing words, we used the familiar language paired with the French language to facilitate memorization. On this basis, English, the subjects’ second language, was chosen as the pairing language, taking into account that Chinese and French are not cognate languages and that the subjects may have associative memory during the memory process.

**Figure 1:**
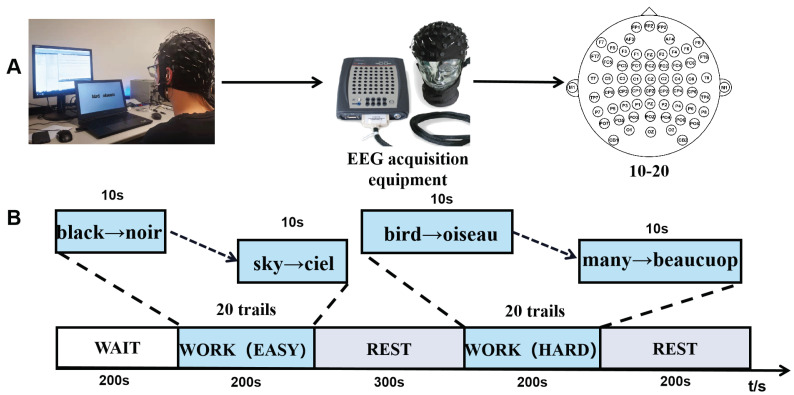
The working memory paradigm. (**A**) The EEG acquisition equipment. The electrode cap conforms to the international 10–20 standard. (**B**) Working memory experimental paradigm framework. The French word from the easy and hardworking memory task, are selected based on the recommendations of linguists to make sure the subjects experience a different working memory load.

The experiment paradigm is shown in Figure 1. Initially, subjects will wait for 200 s to calm down, and it could reduce the effect of stress on the results of the experiment. Then, the memory tasks are carried out. The experiment contains two memory tasks. According to the previous study [34], We separate the different difficulties of working memory by the amount of memory load. The first memory task is easy, and the second task is hard. Each task includes 20 trails and needs 200 s to complete (Figure 1B). At the end of each memory task, All subjects are tested to check their working memory capacity and to ensure that they take the experiment seriously. After the first task end, the subjects will have 200 s to rest until the next work task starts. The word-based visual stimuli are generated by E-Prime software.

### 2.2. Subject Qualification

We invited 20 right-handed students as subjects (10 males and 10 females; mean age: 22 ± 2.6 standard deviations). All subjects report no brain injury and medical treatment history, no implanted medical device in the body, no neurological disease family history, and pregnancy. The Ethics Committee of the Brain Hospital Affiliated of the Nanjing Medical University approved the experimental protocol [2020-KY052-01]. This protocol complies with the Ethical Principles for Medical Research Involving Human Subjects established by the Declaration of Helsinki. In the two weeks after the experiment, we did not receive any adverse feedback.

### 2.3. EEG Recording

EEG recording is obtained from the scalp by 64 Agcl electrodes (curry7 system, $NEUROSCAN^{\mathrm{TM}}$) embedded in quick-cap. The electron position determined by international standard 10–20 Figure 1A. The electrooculogram (EOG) is recorded by four additional electrons, the vertical EOG located on the left eye up and down and the horizontal EOG lateral to the left and right canthus. The M1 and M2 are set as reference electrodes. To make sure the impedance is below 10 kΩ, the electrolyte gel will be inserted into the electrodes. Quick-cap is connected with the amplifier (sample rate: 500 Hz, high pass: 0.5 Hz, low pass: 200 Hz, accuracy: 0.15 nV/LSB) produced by $NEUROSCAN^{\mathrm{TM}}$.

## 3. Method

### 3.1. Algorithm Configuration

**Figure 2:**
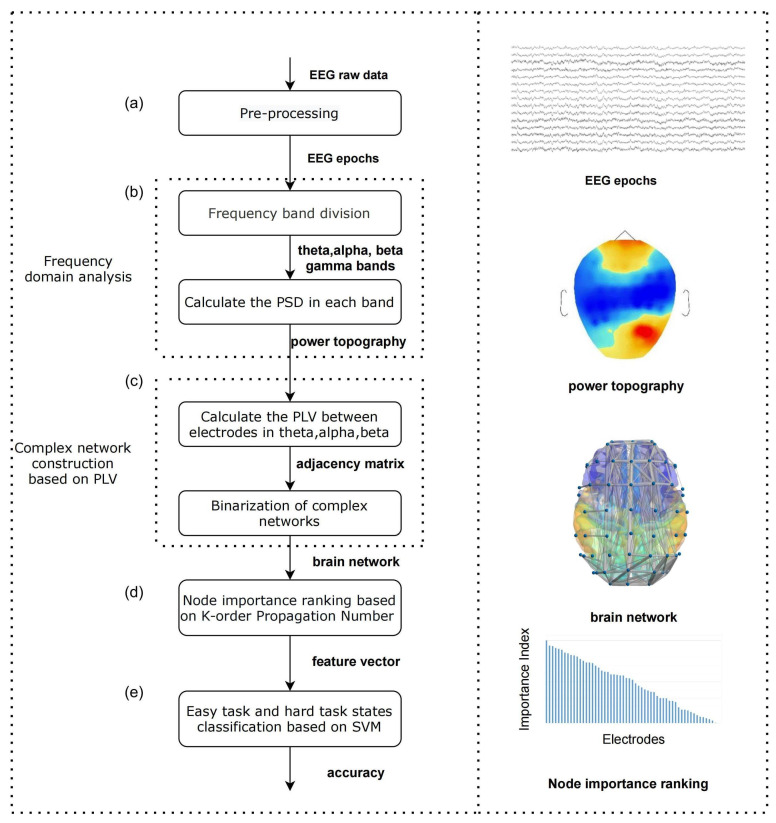
Algorithm configuration. The data processing steps in the algorithm were shown as (**a**–**e**). (**a**) Pre-processing the raw EEG data. (**b**) Perform band division and power spectrum estimation on the processed data. (**c**) Calculate the phase-locked values between EEG channels in the interested frequency bands to construct a normalized brain network. (**d**) Node importance analysis based on the brain network. (**e**) Classification of working memory states based on node importance features of brain networks. The visual diagram of the processed data is shown at the right. EEG epoch is obtained after pre-processing. The power topography is generated by the PSD in theta band. The brain network is obtained based on the PLV adjacency matrix binarization, and the reasonable selection of the threshold value can effectively distinguish the networks with large differences in weights.

A new algorithm called WKPN is applied to analyze the mechanism of working memory by calculating the ranking of node importance which as the feature vector support the classification of working memory states for different memory loads (easy and hard task). The algorithm configuration is shown in Figure 2. A series of steps are summarized as follows: (a) Pre-processing. The raw EEG recordings x(t) are filtered and theirs artifacts are removed to obtain the clean signal epoch A(t). (b) Frequency domain analysis. The spectrum is divided into theta, alpha, beta, and gamma bands, the EEG epochs under the different different WM states(easy state and hard state) are analyzed in each band by power spectrum density (PSD) to explore the spectrum characteristic associated with the memory load. (c) Complex network construction based on Phase Locking Value (PLV). We computed PLV between electrodes to generate the adjacency matrix and binarizing it to construct the undirected, binarized brain network. (d) Node importance ranking based on Weighted K-order Propagation Number (WKPN) algorithm. We proposed K-order Propagation Number algorithm to calculate the nodes importance ranking of the binarized brain network. (e) Different memory load states classification based on Support Vector Machine (SVM). The nodes importance ranking is set as the feature vector of SVM to train the classification results of easy state and hard state.

### 3.2. EEG Data Pre-Processing

**Figure 3:**
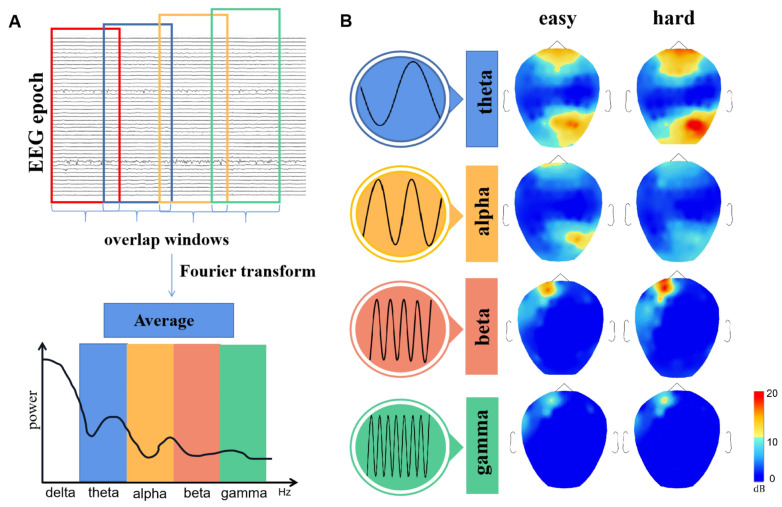
The PSD topographies of four frequency bands in easy task and hard task states. (**A**) Calculating the PSD with the Welch method and the performance of spectral estimation is improved by overlap window method. (**B**) The PSD topographies in easy task and hard task states, the average power is drawn at each frequency band. The alpha power change is mainly located at the posterior area of the brain, especially at the right hemisphere (p=0.067). Comparing with the easy task state, the power amplitude of beta in the working state at the left prefrontal area improved significantly (paired-samples test: p=0.01<0.05). The theta band power amplitude improved at the prefrontal and right posterior area in both easy and hard states, and a more significant rise of amplitude is found in hard state.

The signal pre-processing module is implemented using the MATLAB^®^ (The Math Works Inc., Portola Valley, CA, USA), including two functions with Brainstorm, EEGLab [35,36] Figure 3A. The band-pass filter with high pass 0.5 Hz and low pass 60 Hz is used [37], and the 50 Hz notch filter is applied to remove the power line interference. The eyes movements could be removed by EOG recordings. The other artifact includes Electromyographic (EMG) and ECG are removed by Independent Component Analysis (ICA). M1 and M2 as the reference electrodes are removed. After the pre-processing, the EEG signal is segmented into 10 s epochs for further analysis (baseline corrected for each output file).

### 3.3. Frequency Domain Analysis

To begin with, all epoch signals are transformed from time domain sequences to the frequency domain spectrum. In addition, the spectrum is split into four bands with respect to international standard: theta (3–7 Hz), alpha (7–13 Hz), beta (13–30 Hz), gamma (30–60 Hz). Considering that the power spectrum in the delta band (1–3 Hz) is not strongly correlated with the cognitive processes of working memory [38], no further research was done here. Next, we used the Welch method to calculate the PSD for each band. The point-by-point paired-samples test (95% confidence interval level) is applied for searching the bands and brain regions which demonstrate noticeable variation in PSD between easy task and hard task states.

The result of the brain topographies in four frequency bands (theta, alpha, beta, gamma) is shown as Figure 3, the alpha power of the right posterior area is lower in the hard task state, indicating the alpha oscillation has been inhibited in working memory (similar with [39]). It is might associated with the arrangement of cognitive resources. The amplitude of beta power at the left prefrontal was observed to increase with memory load. The similar phenomenon is also found in theta band, that the power at the frontal and right posterior area is higher in hard task state (similar with [40]). Besides, the right posterior area in the theta band is also activated in the two states, especially in the hard task state. This phenomenon is probably caused by the visual stimuli in our paradigm. It means the posterior area is activated to process the visual signal, and then the prefrontal area deals with the cognitive information in working memory tasks. Considering that the PSD distribution in the gamma band is similar to its in the beta band, but the change between the two states is not significant in the gamma band, and the following PLV calculation in the gamma band is too low. based on this, we consider to focus on the theta, alpha, beta bands in the following PLV brain network construction.

### 3.4. Complex Network Construction Based on Phase Lock Value

**Figure 4:**
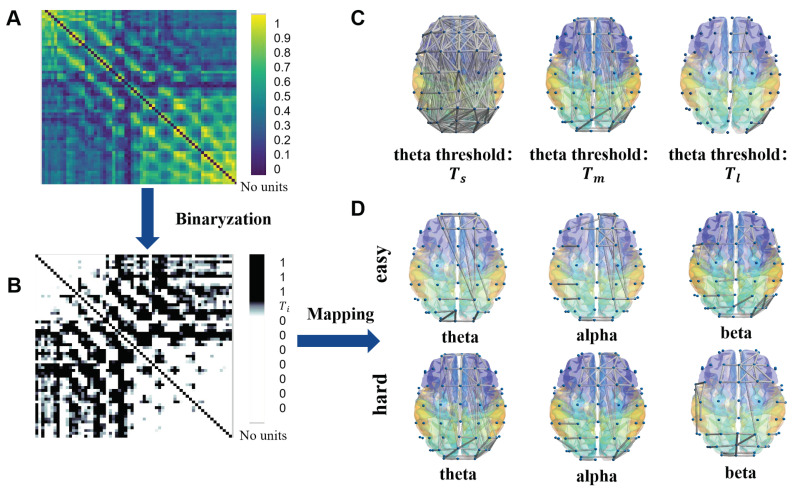
The binarization of the brain network. (**A**) The adjacent matrix of the PLV generated by a group of sampled data. (**B**) The binarized adjacent matrix. The PLV between two electrodes will be 1, if it exceeds the threshold $T_{i}$ and 0 if it is below the threshold $T_{i}$. (**C**) The brain network in the theta band is constructed under different threshold. The edge between the nodes are drawn when the PLV exceed the threshold. The diagram is drawn by the Brain Net Viewer [41]. Considering the PLV will decrease with the frequency increasing, each band must set its own thresholds to establish uniform standard. (**D**) The binarized brain network under the easy task and hard task states. In each band, the thresholds are set individually.

Phase lock value indicated the synchronization of the two signals is used to construct the complex brain networks. At first, the PLV between electrodes in theta, alpha, and beta bands is calculated. Assuming signals of two electrodes are $X_{1}\left(t\right)$ and $X_{2}\left(t\right)$, $Z_{i}$ was its analytic signal, their relative phase Δϕ(t) was computed using Hilbert transform. The formulas of PLV are expressed as:

(1) $$Z_{i}\left(t\right)=X_{i}\left(t\right)+jHT\left(X_{i}\left(t\right)\right),$$

(2) $$\Delta \phi \left(t\right)=arg\frac{Z_{1}\left(t\right)Z_{2}^{*}\left(t\right)}{|Z_{1}\left(t\right)||Z_{2}\left(t\right)|},$$

(3) $$PLV\left(t\right)=|E[e^{j}\Delta \phi \left(t\right)]|,$$

where the $Z_{i}$ is analytic signal, *j* represents the imaginary part, and HT is Hilbert transform which calculates the relative phase ϕ(t); arg calculates phase angle; E|·| represents the expectation of the phase difference between two signals and is used to measure the degree of correlation between the phase changes of the two signals. The PLV is defined from 0 to 1 Figure 4. The higher the PLV value, the stronger phase correlation between signals from two electrodes. Secondly, the PLV between all electrode pairs formed the brain network, in which the nodes correspond to the electrodes and the edge correspond to the PLV. However, the sparsity of the brain networks will change with different edge threshold $T_{i}$ significantly. Figure 4C exhibits the connectivity of the brain network under different edge thresholds in the theta band $\left(T_{l}>T_{m}>T_{s}\right)$. With the increasing of threshold, the brain network structure became sparseness. To reduce computational complexity and establish uniform standard, the threshold should be set in each band to construct the binarized brain network.

Then, the binarization of brain network in each band is achieved according to the surrogate data methods. Hurtado et al. [30] generates new data with the same power spectrum, but without the linear characteristics like the original data, exhibit the difference in weights of functional networks excellently.

The binarized brain network in three bands are shown as Figure 4. In the theta band, the rhythm synchronization between the left frontal and parietooccipital area is increased significantly in the hard task state (p=0.035⩽0.05), which indicates the left frontoparietal network is activated that may be associated with word memory in our paradigm. According to the previous researches, the frontoparietal network was considered as the network related to language processing [42]. In the alpha band, connectivity of the left and right frontal was enhanced in the hard task state. Meanwhile, the connectivity in the beta band is also reinforced at the left temporal lobe in the hard task state.

According to the above result, we could observe that the binarized brain network can clearly demonstrate the difference in connectivity of the brain network between the different memory load states. Next, we will use a new algorithm to further investigate the characteristics of binarized brain networks.

### 3.5. Node Importance Based on K-Order Propagation Number Algorithm

K-order Propagation Number proposed by our team as a novel algorithm, and be used to explore the nodes importance of PLV brain networks in this study [31,43]. The algorithm is inspired from the disease propagation model, abstracts each node as an infectious source, and by comparing the number of infected nodes in the network after the same propagation time K, the larger the number, the more important the node.

The specific node importance is calculated as follows: According to the previous binarized brain network, the $V=\{v_{1},v_{2},v_{3},\ldots ,v_{n}\}$ was set as the nodes, total 62 nodes (n = 62), $e_{ij}$ represent the edge between $v_{i}$ and $v_{j}$.

**Step 1**: Calculating the K -order propagation number. In the brain network, we set the $v_{i}$ as a source of infection, the number of the infected nodes after K propagation time is defined as K-order propagation number $N_{v_{i}}^{K}$.

(4) $$N_{v_{i}}^{K}=\sum_{v_{i}\in V}I\left(D\left(v_{i},v_{j}\right)⩽K\right),$$

$D(v_{i},v_{j})$ represents the shortest path from $v_{i}$ to $v_{j}$. I is the indication function. When the shortest path $D(v_{i},v_{j})⩽K$, the indication function I(·)=1, otherwise, I(·)=0. K∈[0,d], *d* is the maximum connected path of the network.

**Step 2**: Testing the heterogeneity of the network. To reduce the effect of different propagation time K, the structure entropy $H^{k}$ is calculated to measure the heterogeneity of the network. The larger of the $H^{k}$, the less the difference between the nodes’ importance.

(5) $$H^{K}=-\sum_{i=1}^{n}\frac{N_{v_{i}}^{K}}{\sum_{j=1}^{n}N_{v_{j}}^{K}}log\left(\frac{N_{v_{i}}^{K}}{\sum_{j=1}^{n}N_{v_{j}}^{K}}\right),K\in \left\{0,1,\ldots ,d\right\},$$

**Step 3**: Calculating the ranking of the node importance in the brain network. The propagation time play an important role in the algorithm. If the importance of a node is measured only by the number of infected individuals in the network at a single propagation time, it may miss information at other propagation times. Therefore, in this paper, all moments of K taken from 0 to d will be examined together. The importance of the nodes is calculated according to the structure entropy $H^{k}$ and the K-order propagation number in different K value.

(6) $$Q_{v_{i}}=\sum_{K=0}^{d}c^{K}\;\cdot \;S_{v_{i}}^{K},$$

where $c^{K}$ and $S^{K}$ are the normalized results of $H^{K}$ and $N^{K}$. The ranking of the important nodes $Q_{v_{i}}$ shows the characteristic of brain network in two different work load states.

### 3.6. Different Memory Load States Classification Based on SVM

The SVM was aim to find a hyperplane that can correctly separate the two types of data points as much as possible [44]. In this study, the SVM will classify the the different memory load states according to the node importance characteristics of the brain network. The 62 nodes importance ranking of 20 subjects provide 800 group data for training. Considering that the sets of node importance in the easy task and hard task states may partially overlap, the linear SVM is not applicable here. A divisible SVM solves this difficulty, is made linearly divisible in Hilbert space by transforming the training set into a high-dimensional Hilbert space where make the set is linearly divisible. At the same time, we intend to reduce the number of extracted feature nodes as much as possible based on ensuring the classification accuracy.

## 4. Result

Based on the WKPN algorithm, 1600 epochs from 20 subjects are used to calculate the node importance features of the brain network, which are set as the feature vector of the SVM. The feature vectors of all samples are randomly assigned as training and test samples in the ratio of 3:2 for cross-validation to ensure that the data samples are randomly assigned for cross-validation. We repeat this process several times to ensure that the assignment of samples is random and different each time.

### 4.1. The Nodes Importance of Brain Network

**Figure 5:**
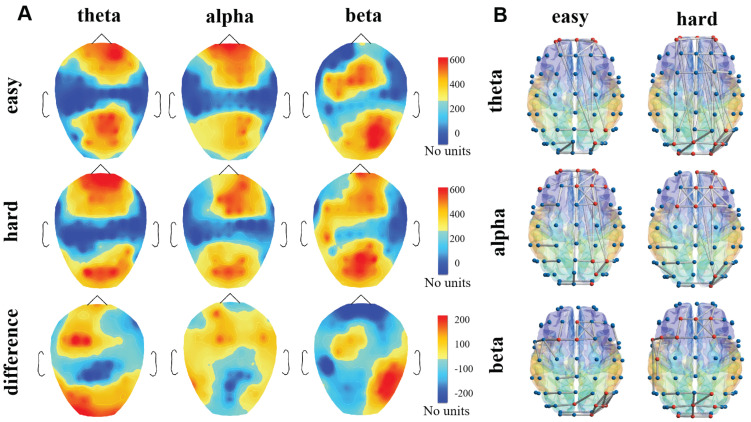
The topography diagram of the node importance distribution. The diagram of three bands is drawn according to the result of the K-order propagation number algorithm. (**A**) Brain topography of node importance distribution. (**B**) The brain network topology diagram. The value of the node importance is mapped from 0 to 1, the node with the importance exceed 0.8 is marked as red.

Figure 5 reveals the distribution of nodes importance in PLV brain networks. As the memory task load increasing, the distribution of important nodes also changes. In the theta band, the important nodes are mainly located at the frontal and occipital lobe, which consistent with the results of 3.3 where the frontal and occipital lobes were activated by working memory. As memory load increasing, important nodes were transferred from the right prefrontal to the frontal midline. The difference in the distribution of important nodes under the two states is shown in Figure 5A, the importance of nodes (FC3, PZ, O1) exist significant difference (*p* < 0.05). Contrary with the theta band, the location of the important nodes transforms from the frontal midline to the right front-parietal lobe in alpha band, and the difference of node importance between the two states was reveled at the node POZ (*p* = 0.03 < 0.05). In the beta band, we can observe the significant change (*p* < 0.05) in the distribution of brain network node importance at the left temporal parietal lobe TP7 and right posterior occipital lobe PO4 in the two states.

To explore the changes in the importance of brain network nodes under different memory loads more objectively, we normalized the node importance index, set the same threshold, and the nodes whose importance exceeded the threshold would be marked. By examining the number of marked nodes in different states, we can conclude that the number of important nodes increases with memory load in the theta and beta bands, and thus the synchronization of the brain network increases with the memory task. In contrast, the number of marked nodes in the alpha band decreases. In summary, the results of K-order propagation number can well distinguish the characteristics of brain networks in different memory load states.

### 4.2. The Classification Accuracy of SVM

**Figure 6:**
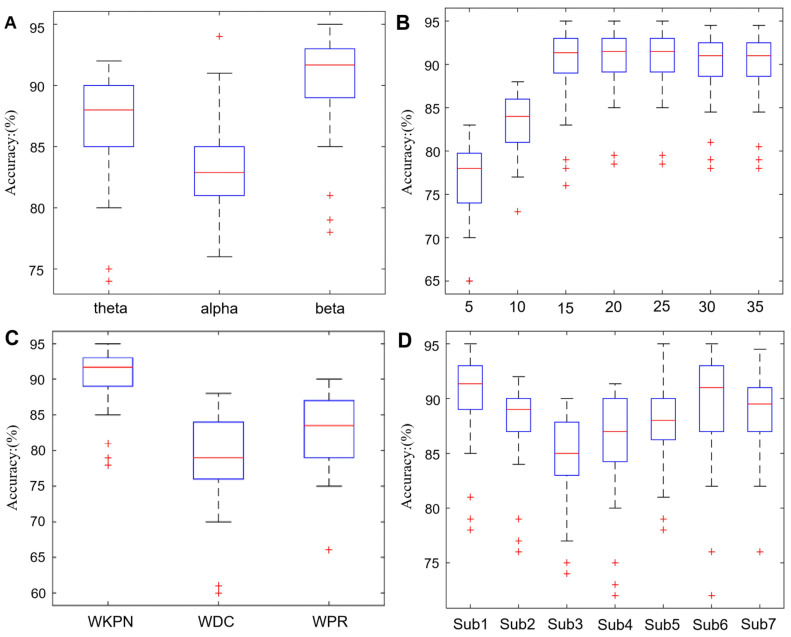
The classification accuracy rate. (**A**) The classification accuracy results of three bands. The classification accuracy is range from 80% to 92% in theta band, 72–91% in alpha band, and 85–95% in beta band. The highest mean classification accuracy is 92% achieved in beta band. (**B**) The beta band classification accuracy with different length feature vector. (**C**) Comparison of classification accuracy between WKPN, WDC, and WPR algorithm. (**D**) Classification accuracy differences between subjects in the beta band. Each ‘+’ stands for one outlier result of this group.

The classification results of SVM are shown in Figure 6. The classification accuracy of three bands approximate range from 72% to 95% Figure 6A, and the highest mean classification accuracy is achieved in beta band (mean accuracy theta: 87%, alpha: 82%, beta: 91%), so, the beta band is set as reference band for WM state classification.

Meanwhile, in order to save the computing time while ensuring the accuracy, according to the ranking of the node importance, the classification accuracy with the different length of feature vectors in the beta band is calculated. Figure 6B shows that, initially, the classification accuracy increases as the length of the feature vector grows, however, as the vector length continues to grow, the classification accuracy may decrease due to the local optimal solution, and the classification accuracy is highest when the feature vector containing 20 elements. Through this procedure, the running time of the algorithm is reduced by 10 s.

To further verify the classification effectiveness of the algorithm, we used the WDC and WPR algorithms to analyze the node importance of the brain network in the beta band, and the network features of two algorithms were used as input data for the SVM. The number of training times and samples are the same as those of SVM. Figure 6C reveals that the SVM which use the WKPN algorithm to calculate importance of brain network nodes as the feature vector has higher classification accuracy. Also, we calculated the classification accuracy in the beta band between subjects, and the results ranged from 77% to 95%. This may be due to differences in signal quality as well as in the working memory capacity of subjects. This requires more care in the acquisition of data and pre-processing to minimize this effect.

## 5. Discussion and Conclusions

In this paper, we proposed an original experimental paradigm grounded on word memory to scrutinize the mechanism of the WM from an EEG angle. To begin with, PSD is harnessed to inspect the power attribute of the theta, alpha, beta, and gamma bands through the easy and hard task; here we concentrate on the bands with substantial power differences between the two states. Additionally, the complex brain networks are set up by taking advantage of PLV, which can manifest the properties of rhythm synchronization in the corresponding bands. We eventually operated the K-order propagation number algorithm to compute the node importance of the brain networks.

In contrast to the conventional EEG signal characterization analysis approaches, several merits of this work are underlined as follows: (1) A novel experimental paradigm is proposed to provoke WM by remembering French words. The paradigm is planned in the practice of the English and French word pair, hence it reduces the obstruction of the experimental results by the subjects with language bias. Since there are a uncommon studies emphasis on WM of words, our paradigm supplements the researches in this area. (2) Studying WM from the view of brain networks blended with conventional time-frequency domain methods. Seeing that WM is a joint process between several brain regions and based on accessible studies, WM is allied with the rhythm synchronization among diverse brain regions, we can well uncover the features of EEG signals during WM by constructing a PLV based brain network: the rhythm synchronization between the prefrontal and the parietooccipital rises when increasing WM load. (3) We offered a novel algorithm, i.e., the K-order propagation number algorithm. To study the characteristic of brain network, WKPN, Compared with other algorithms, takes into account the function of bridge nodes in the network, which has less computational complexity and superior robustness. The calculated node importance sequences can well bare the characteristics of the brain network under altered memory loads, which we set as feature vector as the input of the SVM, which can achieve about 95% accuracy in the beta band.

Nonetheless, limitations exist and our work needs to be improved: (1) The capacity of WM is correlated with the rhythmic synchronization of neuroelectric activity. However, the neuronal rhythm synchronization is influenced by one brain region to another. Consequently, we should construct directed brain networks in future research, which can make the outcomes more valid. (2) The heterogeneity among subjects is omnipresent in the task of classification, which can be associated to our setting of thresholds when normalizing the brain network. As a result, extra fine-tuning of the algorithm is indispensable.

In the future, the succeeding issues will be addressed: (1) The directed brain network construction. Regarding the Granger causality between nodes, it is feasible to perceive the movement of information amid different brain regions throughout WM tasks and could better consider the node importance of the brain network. (2) Devise a transcranial stimulation paradigm that can be adaptively regulated based on the node importance analysis of brain networks. Recently, Transcranial Electric Stimulation (tES) method could augment the connectivity between the neurons by applying micro-electrical stimulation at specific brain regions shed lights into neuroregulation and treatment field [45], in which the transcranial Alternating Current Stimulation (tACS) demonstrate properties that can regulate the rhythmic activity of the brain in specific frequencies [46]. Yet, the implementation of tACS also requests to consider the task-related rhythm synchronization frequency and the associated brain regions [47]. To solve this problem, we decided to study the important nodes in the brain network during working memory from the perspective of the brain network to determine the stimulation location of the tACS, and through the time-frequency analysis of the EEG signal at the related brain regions to ascertain the corresponding stimulation frequency. The stimulation paradigm could achieve adaptive modulation for various tasks and subjects. This transcranial stimulation paradigm has yielded good results in our current study, and we hope to keep refining this research.

## Data Availability

Not applicable. However, for academic purposes and justified reseaons, it can be obtained by contacting corresponding author.
